# Exploring Gene Expression and Alternative Splicing in Duck Embryonic Myoblasts via Full-Length Transcriptome Sequencing

**DOI:** 10.3390/vetsci11120601

**Published:** 2024-11-27

**Authors:** Jintao Wu, Shuibing Liu, Dongcheng Jiang, Ya’nan Zhou, Hongxia Jiang, Xiaoyun Xiao, Boqian Zha, Yukai Fang, Jie Huang, Xiaolong Hu, Huirong Mao, Sanfeng Liu, Biao Chen

**Affiliations:** 1 College of Animal Science and Technology, Jiangxi Agricultural University, Nanchang 330045, China; wjt884007846@stu.jxau.edu.cn (J.W.); 18879138275@163.com (S.L.); jdc1701136202@stu.jxau.edu.cn (D.J.); zhouyanan@stu.jxau.edu.cn (Y.Z.); 13340646380@stu.jxau.edu.cn (H.J.); xiaoxiaoyun@stu.jxau.edu.cn (X.X.); fuchun@126.com (B.Z.); 18079769256@163.com (Y.F.); 15679165079@163.com (J.H.); huxiaolong@jxau.edu.cn (X.H.); huirongm@jxau.edu.cn (H.M.); 2 Poultry Institute, Jiangxi Agricultural University, Nanchang 330045, China

**Keywords:** duck, skeletal muscle, full-length transcriptome, differentially expressed genes

## Abstract

The duck industry is vital for supplying high-quality protein, making research into the development of duck skeletal muscle critical for improving meat and egg production. In this study, we leveraged Oxford Nanopore Technologies (ONT) sequencing to perform full-length transcriptome sequencing of myoblasts harvested from the leg muscles of duck embryos at embryonic day 13 (E13), specifically examining both the proliferative (GM) and differentiation (DM) phases. Our analysis identified a total of 5797 novel transcripts along with 2332 long non-coding RNAs (lncRNAs), revealing substantial changes in gene expression linked to muscle development. We detected 3653 differentially expressed genes and 2246 instances of alternative splicing, with key genes involved in essential pathways, such as ECM–receptor interaction and Notch signaling, prominently featured. Additionally, we constructed a protein–protein interaction network that highlighted critical regulators—*MYOM3*, *MYL2*, *MYL1*, *TNNI2*, and *ACTN2*—associated with the processes of proliferation and differentiation in myoblasts. This extensive transcriptomic investigation not only sheds light on the intricate molecular mechanisms driving skeletal muscle development in ducks but also provides significant insights for future breeding strategies aimed at enhancing the efficiency of duck production. The results emphasize the efficacy of ONT sequencing in uncovering complex regulatory networks within avian species, ultimately contributing to progress in animal husbandry.

## Simple Summary

The aim of this study was to analyze alternative splicing events and differentially expressed genes in duck embryonic myoblasts using Oxford Nanopore Technology (ONT) sequencing, focusing on both proliferating and differentiating myoblasts. We sought to elucidate the molecular mechanisms involved in duck skeletal muscle development. We identified a total of 5797 novel transcripts along with 2332 long non-coding RNAs (lncRNAs). We also detected 3653 differentially expressed genes and 2246 instances of alternative splicing. These findings highlight the complexity of gene expression and its crucial role in diverse cellular functions and reveal substantial changes in gene expression linked to muscle development. An enrichment analysis revealed several key genes that are closely linked to skeletal muscle growth and development, positioning them as strong candidates for regulating the proliferation and differentiation of duck embryo myoblasts. Overall, these findings enhance our understanding of the molecular mechanisms driving muscle development in ducks and highlight potential strategies for improving meat quality and production traits through targeted genetic interventions.

## 1. Introduction

Ducks are of significant economic importance as poultry. Their lineage is traced back to mallards in central China around 500 B.C. Renowned for its high protein levels and nutritional benefits, duck meat enjoys widespread popularity in the global meat market [1,2]. Skeletal muscle makes up roughly 40–50% of a duck’s body weight [3] and is the primary source for meat production. The development of skeletal muscle occurs through a complex series of stages, divided into three major phases. The initial phase involves the formation of embryonic muscle fibers; this includes the proliferation and differentiation of myoblast precursors, the fusion of these myoblasts into multinucleated myotubes, and the maturation of myofibrils. The next phase focuses on the development of embryonic myofibers, marked by changes in various fiber types. The final stage pertains to adult muscle regeneration, which involves increases in myofiber diameter and length, as well as the repair of damaged myofibrils [4]. The first stage is especially critical, as this ultimately determines the number of muscle fibers produced [5].

The intricate mechanisms governing skeletal muscle development are regulated by numerous genes. Muscle regulatory factors (MRFs) are essential in myogenesis, orchestrating the differentiation of myoblasts into myotubes, facilitating myofiber formation, and managing the expression of muscle-specific genes [6,7,8]. Additionally, epigenetic modifications significantly contribute to the growth and development of skeletal muscle. This encompasses processes such as DNA methylation, histone modification, RNA editing, and the involvement of non-coding RNAs like microRNAs, long non-coding RNAs (lncRNAs), and circular RNAs (circRNAs) [9,10,11]. Although considerable progress has been made in understanding skeletal muscle biology, the distinct regulatory pathways involved in muscle development in ducks—a non-traditional model organism—remain underexplored.

Oxford Nanopore Technology (ONT) sequencing is an advanced method that employs nanopore-based technology to achieve single-molecule, real-time sequencing by detecting electrical signals from nucleic acids as they pass through a protein nanopore [12,13]. This cutting-edge approach offers longer read lengths, immediate data generation, and direct RNA sequencing capabilities, thus revolutionizing transcriptomics research [12,14]. ONT sequencing has proven to be beneficial in various studies related to livestock and poultry, including investigations into the complexity of the pig transcriptome [15], the identification of genes linked to follicular selection in chickens [16], and comparative analyses of transcriptomes across different bovine tissues [13]. However, reports on utilizing ONT sequencing for studying duck embryo skeletal muscle are currently lacking.

In this research, we isolated myoblasts from the leg muscles of duck embryos at embryonic day 13 (E13). We examined two different stages. One group was collected immediately following three differential attachment stages to capture proliferating myoblasts (GM); the other group consisted of cells that were cultured in a differentiation medium for 48 h following successful isolation, including a substantial number of formed myotubes (DM). By applying ONT sequencing, we obtained full-length transcriptomes for both proliferating and differentiating myoblasts. Our analysis of alternative splicing events and differentially expressed genes in duck embryonic myoblasts helps to clarify the molecular mechanisms involved in skeletal muscle development. Furthermore, these findings will lay the foundation for clarifying the development and regulatory mechanisms of skeletal muscle in duck embryos.

## 2. Materials and Methods

### 2.1. Isolation and Culture of Cells, Along with Sample Collection

Duck eggs used in this investigation were obtained from breeder ducks raised at Jiangxi Agricultural University’s Poultry Research Institute. It has been shown that duck embryos at embryonic day 13 are more likely to isolate duck embryo myoblasts and have undifferentiated cells [17]. The incubation process was conducted until embryonic day 13, at which point primary myoblasts were extracted from the leg muscles. Detailed methodologies for egg incubation and muscle cell isolation can be found in our earlier publications [18]. Briefly, leg muscle tissues were carefully dissected and minced into fine pieces. The tissue was then subjected to digestion using 0.25% trypsin-EDTA (Gibco, Waltham, MA, USA) at 37 °C for 15 min, after which fetal bovine serum (FBS) (Gibco) was introduced to halt the enzymatic activity. The resulting cell suspension was filtered through a mesh and centrifuged at 1500 rpm for 5 min to collect the myoblasts. To ensure the removal of fibroblasts, the cells underwent three rounds of differential attachment, allowing only non-adherent myoblasts to remain. Following another centrifugation step, a portion of the collected cells was lysed in 1 mL of TRIzol (Invitrogen, Carlsbad, CA, USA) and stored at −80 °C, while the remainder was resuspended in a complete medium comprising 15% FBS and RPMI 1640 (Gibco), and cultured in 60 mm tissue culture dishes. Once the cultures reached approximately 90% confluence, the medium was switched to RPMI 1640 supplemented with 2% horse serum (Gibco) and 0.5% penicillin/streptomycin (Solarbio, Beijing, China) to induce differentiation in the myoblasts. Cells were harvested on the second day of the differentiation process. Myoblasts collected immediately after the third round of differential adhesion were classified as the proliferating myoblast group (GM), while those collected after 48 h in the differentiation medium were classified as the differentiated myoblast group (DM).

### 2.2. Construction and Sequencing of Full-Length cDNA Libraries

The concentration and quality of RNA were evaluated using a NanoDrop UV spectrophotometer (NanoDrop, Carlsbad, CA, USA) and a Qubit 3.0 Fluorometer (Life Technologies, Carlsbad, CA, USA). RNA integrity was assessed via agarose gel electrophoresis. After quality was confirmed, complementary DNA (cDNA) synthesis was executed. A long-read cDNA library was constructed utilizing the ONT PCR amplification cDNA kit (SQK-PCS109, Wuhan, China). Specifically, full-length cDNA libraries were generated from poly(A)+ mRNA using a dedicated cDNA PCR sequencing kit. Following this, 13 to 14 cycles of PCR amplification were completed with Oxford Nanopore PCR adaptor kits (SQKPBK004, Wuhan, China). The cDNA was then ligated with 1D sequencing adaptors and loaded onto R9.4.1 FLO-PRO002 nanopores for sequencing on the PromethION platform (ONT, Wuhan, China).

### 2.3. Quality Control for Sequencing Data

Raw sequencing data from ONT were subjected to quality control using NanoFilt (v2.8.0). Sequences with quality scores below 7 and lengths shorter than 50 bp were filtered. Pychopper (v2.4.0) software was employed to discern full-length sequences within the effective data, followed by the alignment of the filtered full-length sequences to the duck reference genome (https://ftp.ensembl.org/pub/release-112/fasta/anas_platyrhynchos_platyrhynchos/dna/ (accessed on 6 July 2024)) using minimap (v2.17-r941).

### 2.4. Sample Consistency Analysis

The rapid construction of a non-redundant transcript set from full-length sequences was performed using Pinfish (v0.1.0), yielding consistent sequences. These sequences were aligned to the reference genome, and redundancy was eliminated from the alignment results using StringTie (v2.1.4). This step allowed for the merging of alignments differing only at the 5′ ends of exons, resulting in non-redundant transcript sequences. Additionally, gffcompare (v0.12.1) was utilized to compare non-redundant transcripts against known transcripts in the genome, facilitating the identification of novel transcripts and genes while improving existing annotations.

### 2.5. Alternative Splicing Analysis

Gene transcription generates precursor mRNA (pre-mRNA), which undergoes various splicing events, selecting different exons to produce distinct mature mRNAs [19]. This mechanism contributes to phenotypic diversity. To identify alternative splicing types present in each sample and determine inter-group differences in splicing, Suppa2 [20] software was utilized.

### 2.6. Simple Sequence Repeat (SSR) Analysis

Simple sequence repeats (SSRs), also referred to as microsatellites, consist of repeating units of 1 to 6 nucleotides, forming repetitive sequences that may extend over several dozen nucleotides. Due to variations in nucleotide composition and differing repeat counts, SSRs exhibit significant variability in length. MISA (v1.0) was employed for SSR predictions [21].

### 2.7. LncRNA Analysis

Predictions regarding the coding potential of newly identified transcripts were made utilizing CNCI (v2.0) [22], CPC (standalone_python3 v1.0.1) [23], and PLEK software. LncRNAs exert regulatory effects primarily through the modulation of target gene expression. Cis-regulation entails lncRNA influence over adjacent mRNAs located on the same chromosome, necessitating the identification of co-located mRNAs for predicting lncRNA target genes. In contrast, trans regulation is independent of positional relationships between lncRNAs and coding genes but relates to co-expression with other genes. The Pearson correlation coefficient method was applied to analyze the correlation between lncRNAs and genes across samples, identifying genes with a correlation coefficient greater than 0.9 as potential targets of the lncRNAs.

### 2.8. Differential Expression Analysis and Functional Annotation

The analysis of differential gene expression was performed using DESeq2 (v1.26.0) [24]. This software utilizes statistical methods grounded in the negative binomial distribution to assess differential expression in digital gene expression data. The criteria for screening included thresholds of padj < 0.05 and |log2FoldChange| > 1. Genes that exhibited notable variations in expression levels across different experimental conditions were classified as differentially expressed genes (DEGs). Additionally, distinct transcripts refer to various mRNA isoforms derived from the same gene, with those showing significant expression level differences termed differentially expressed transcripts (DETs). For the enrichment analysis of differentially expressed genes, clusterProfiler (v3.14.3) [25] was utilized. The software calculates p.adjust, which reflects *p*-values adjusted for multiple hypothesis testing. This value ranges from 0 to 1, with lower values indicating more substantial enrichment significance. clusterProfiler was employed to conduct a Gene Ontology (GO) enrichment analysis on the annotated genes, identifying key biological functions, alongside a Kyoto Encyclopedia of Genes and Genomes (KEGG) pathway analysis to elucidate the primary pathways involved in muscle growth and development.

### 2.9. Protein–Protein Interaction Network (PPI)

DEGs were submitted to the STRING database (https://cn.string-db.org/ (accessed on 4 September 2024)) to discover homologous proteins. A protein–protein interaction network was constructed based on the interaction relationships among these homologous proteins. Cytoscape (v3.10.2) was subsequently used for visualizing this interaction network.

### 2.10. Real-Time Quantitative PCR(RT-qPCR) Validation

From the list of DEGs (Table S10), six genes (*JCHAIN*, *F9*, *LOC101800358*, *CA13*, *LOC101796443*, *LOC101792412*) were chosen at random for validation through quantitative reverse transcription polymerase chain reaction (RT-qPCR). *GAPDH*, a primer set derived from previous studies [26], was used as an internal control gene, and other primers were designed using the NCBI Primer Designing Tool (Table 1). Beijing Qingke Biotechnology Co., Ltd. was commissioned to carry out the synthesis of primers. Results were presented as means ± standard error of the mean (SEM), and statistical analyses were conducted using a two-tailed *t*-test. Significance levels were indicated as * for *p* < 0.05, ** for *p* < 0.01, and *** for *p* < 0.001.

## 3. Results

### 3.1. Duck Primary Myoblast Differentiation

**Figure 1:**
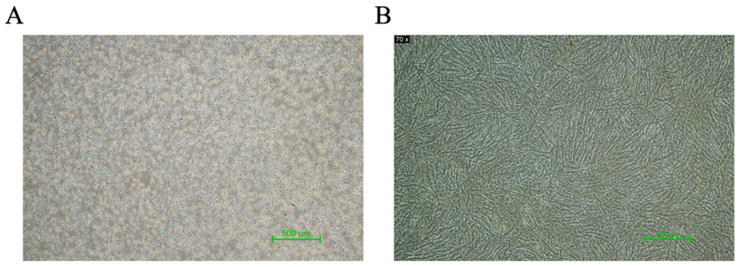
Duck primary embryonic myogenesis. (**A**) Myoblasts after differential adhesion. (**B**) Differentiating myoblasts on 48 h (myotubes).

Leg myoblasts were isolated from 13-embryo-old duck embryos and underwent three differential attachments to obtain proliferating primary myoblasts (GM) with fibroblasts removed (Figure 1A). After inducing differentiation, myotube growth and enlargement were evident at 48 h, as depicted in Figure 1B.

### 3.2. Summary of Full-Length Transcriptome Data

Using the Oxford Nanopore (ONT) platform, we performed full-length transcriptome sequencing across six samples, yielding an average of 3,665,914 clean reads per sample. The overall count of full-length sequences varied between 2,615,122 and 2,905,954, with an average length of 1419 bp. The average N50 of all reads was up to 2011 bp, and the maximum length was 21,953 bp. Notably, more than 75% of these clean reads were categorized as full length, and over 90% of them successfully aligned with the reference genome in all samples (refer Table 2 and Table 3).

### 3.3. Functional Annotation of Novel Transcript

**Figure 2:**
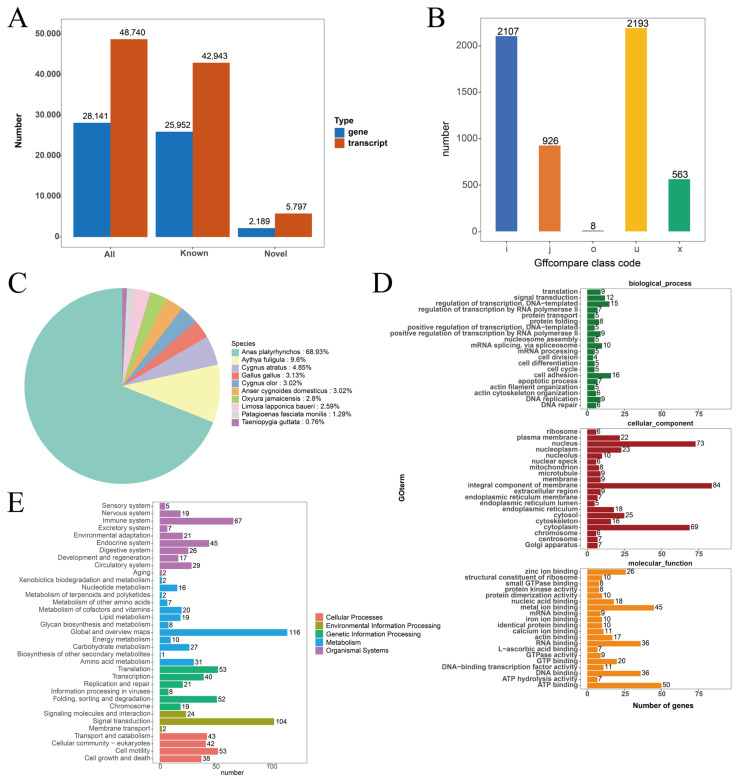
Analysis of new transcripts and variable splicing. (**A**) Number of new transcripts and genes. (**B**) Type and number of novel transcripts. ‘o’ denotes regions on the same strand overlapping with reference exons, ‘j’ signifies at least one matching multi-exon, ‘x’ represents exon overlap on the opposite strand, ‘I’ indicates introns completely contained within the reference transcript, and ‘u’ denotes unknown new transcripts. (**C**) Nr annotation statistics. (**D**) GO enrichment results of novel transcripts. (**E**) KEGG enrichment results of novel transcripts.

A thorough alignment of non-redundant transcripts against established annotations from the duck reference genome (https://useast.ensembl.org/Anas_platyrhynchos_platyrhynchos/Info/Indexin (accessed on 9 July 2024)) led to the discovery of 5797 novel transcripts (Figure 2A). The distribution of these novel transcripts is depicted in Figure 2B. Among these, 1229 were annotated in at least one database, revealing significant entries, including 997 transcripts identified in the KEGG database, 557 in the Pathway database, 1197 in the Nr database, 1216 in Uniprot, 591 in GO, and 25 in KOG (Table 4). Specifically, the Nr database annotation showed that 639 transcripts matched with green-headed mallards, 89 with crested ducks, and 45 with black swans (Figure 2C). Further analysis using GO enrichment revealed that these new transcripts were mainly linked to cellular components like the nucleus, integral membrane components, and cytoplasm. In terms of molecular function, there was notable enrichment for metal ion binding and ATP binding (Figure 2D). Additionally, KEGG pathway analysis indicated significant involvement in pathways associated with the immune system, general overview maps, and signaling processes (Figure 2E).

### 3.4. Alternative Splicing

**Figure 3:**
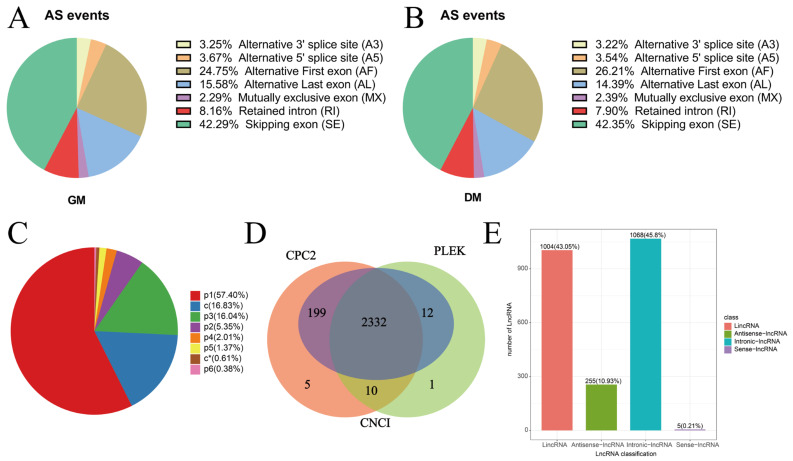
Alternative splicing types. SSR analysis and lncRNA identification. (**A**) Average percentage of each type of alternative splicing in GM samples. (**B**) Average percentage of each type of alternative splicing in DM samples. (**C**) Number and type of SSRs. (**D**) Venn diagram of predicted lncRNAs by the CNCI, CPC, and PLEK. (**E**) Statistics of lncRNA number.

The investigation uncovered 2246 differentially expressed alternative splicing (AS) events, of which 203 were statistically significant (Supplementary Tables S1–S7). The most common AS types included skipping exon (SE) and alternative first exon (AF) events, which were particularly prominent in GM and DM samples (Table 5; Figure 3A,B).

### 3.5. SSR Analysis

In the analysis of SSRs, a total combined sequence length of 85,085,181 bp was examined, resulting in the identification of 15,739 sequences and 28,677 SSR instances (Table 6). These comprised seven categories: single nucleotide, dinucleotide, trinucleotide, tetranucleotide, pentanucleotide, hexanucleotide, and compound SSRs (Figure 3C).

### 3.6. LncRNA Identification

**Figure 4:**
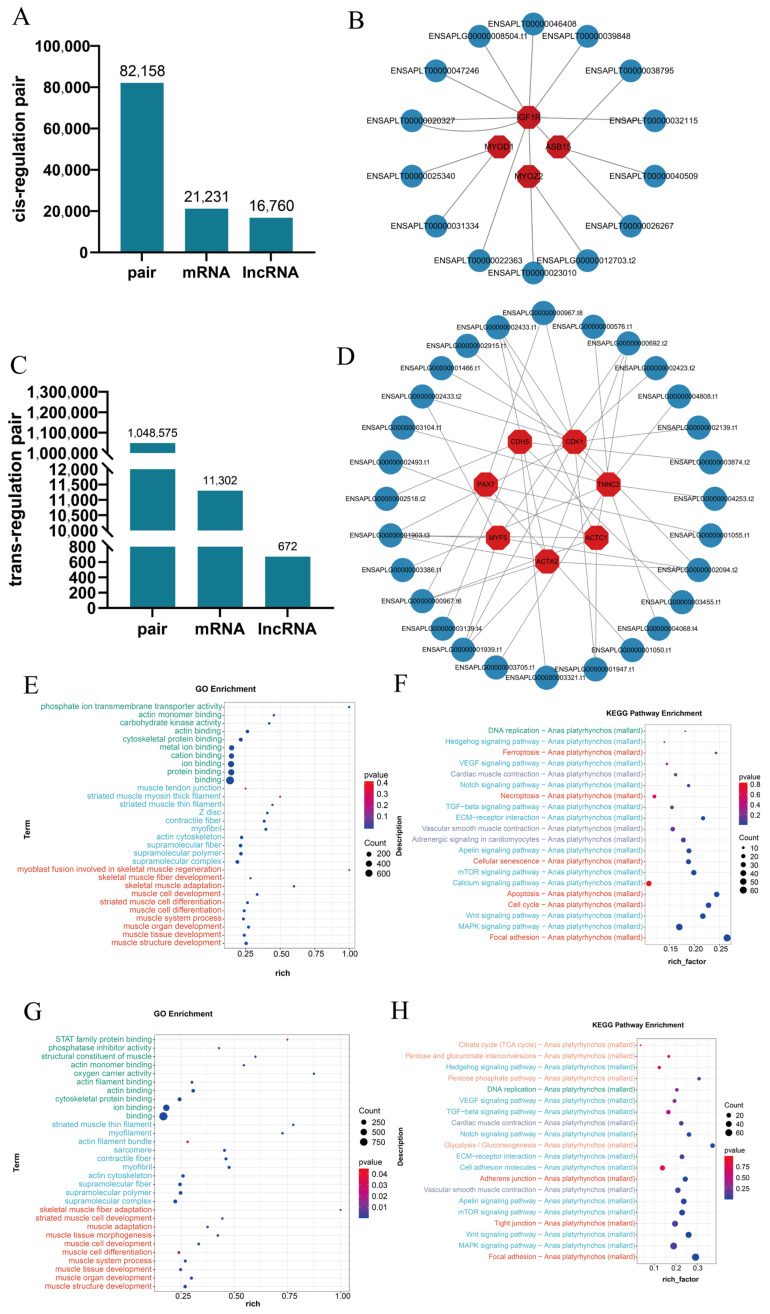
LncRNAs’ function prediction. (**A**) Number of cis-regulated lncRNA mRNA pairs. (**B**) Cis-acting lncRNAs on muscle-related genes. (**C**) Number of trans-regulated LncRNA mRNA pairs. (**D**) Trans-acting lncRNAs on muscle-related genes. (**E**) GO enrichment analysis of the cis-regulated target gene. (**F**) KEGG analysis of the cis-regulated target gene. (**G**) GO enrichment analysis of the trans-regulated target gene. (**H**) KEGG analysis of the trans-regulated target gene.

The methodologies used for lncRNA prediction identified 2332 lncRNAs (Figure 3D). Classification revealed 1004 lincRNAs, 255 antisense lncRNAs, 1068 intronic lncRNAs, and 5 sense lncRNAs (Figure 3E). Moreover, predictions extended to 16,760 lncRNAs, 21,231 mRNAs, and 82,158 cis-regulated lncRNA-mRNA pairs (Figure 4A). Important muscle development-related genes, such as *MYOD1*, *MYOZ2*, *IGF1R*, and *ASB15*, were identified as being cis-regulated by lncRNAs (Figure 4B). Conversely, trans regulation prediction involved 670 lncRNAs, 11,302 mRNAs, and an extensive 1,048,575 trans-regulated lncRNA-mRNA pairs (Figure 4C). ENSAPLG00000001939.t1 can trans-regulate *MYF5*, *CDK1* and *ACTC1*. Other genes under trans regulation included *CDH5*, *ACTA2*, *PAX7* and *TNNC2*, all known for their roles in muscle development (Figure 4D). Both cis- and trans-regulated target genes exhibited notable differential expression (Supplementary Tables S8 and S9). GO enrichment analysis grouped the differentially expressed target genes into three primary categories: biological processes, molecular functions, and cellular components. For both types of regulatory targets, biological process terms emphasized muscle structure development and organ development. Molecular functions highlighted actin binding and structural constituents of muscle, while cellular component terms concentrated on actin binding and actin monomer binding (Figure 4E,G). KEGG pathway analyses further emphasized enrichment in critical signaling pathways related to muscle, such as ECM–receptor interaction, cell-cycle, and MAPK signaling pathways (Figure 4F,H). These results indicate that lncRNAs are vital players in the regulatory mechanisms governing duck embryo myoblast development.

### 3.7. Functional Annotation and Enrichment Analysis of Differentially Expressed Genes

**Figure 5:**
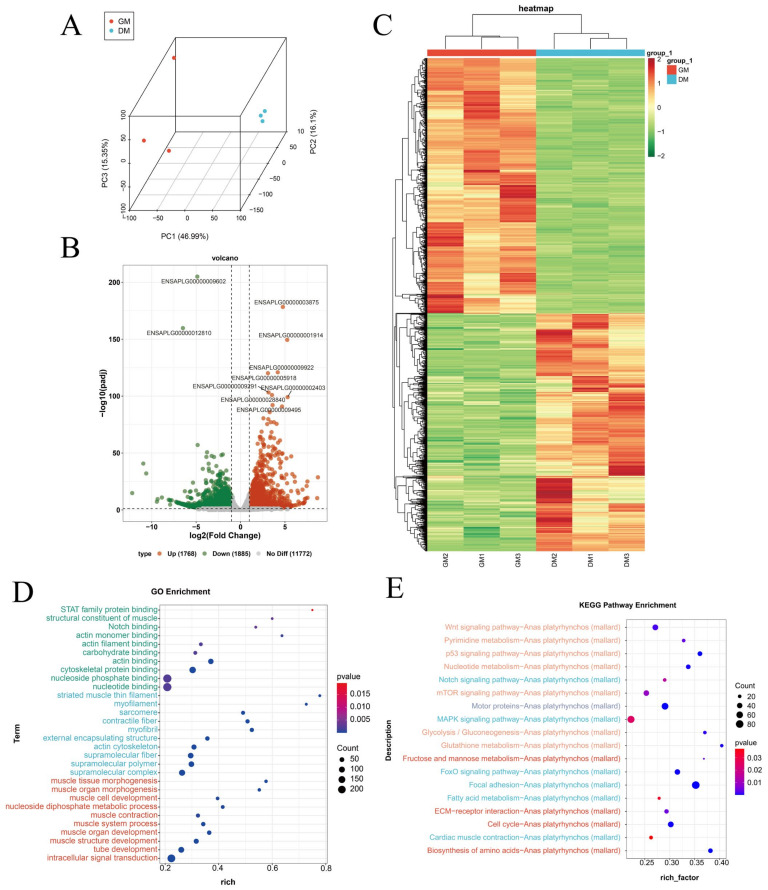
Differentially expressed gene analysis. (**A**) Principal component analysis (PCA) of all samples using sequencing data. (**B**) Differentially expressed gene volcano map. (**C**) Differentially expressed gene heat map. (**D**) Differentially expressed gene GO enrichment analysis. (**E**) Differentially expressed gene KEGG analysis.

Principal component analysis (PCA) revealed distinct clustering between GM and DM conditions, with replicates within the DM group showing strong similarity (Figure 5A). A total of 3653 differentially expressed genes (DEGs) were identified when comparing GM and DM samples, consisting of 1768 upregulated and 1885 downregulated genes (Figure 5B, Supplementary Table S10). Hierarchical clustering based on expression levels revealed clear relationships among the samples and DEGs, illustrated through heatmap representations (Figure 5C). Enrichment analyses of the DEGs revealed significant links to various biological processes, especially those connected with muscle structure development, muscle organ development, tissue morphogenesis, and organ morphogenesis. Cellular composition terms were enriched for elements like myofibrils, contractile fibers, sarcomeres, and the actin cytoskeleton. Furthermore, molecular function terms prominently featured actin binding, structural constituents of muscle, and binding to actin filaments (Figure 5D). KEGG pathway analysis indicated that these DEGs were mainly enriched in the FoxO signaling pathway, along with the cell-cycle, ECM–receptor interaction, and MAPK signaling pathways (Figure 5E).

### 3.8. PPI Network Analysis and Central Gene Identification

**Figure 6:**
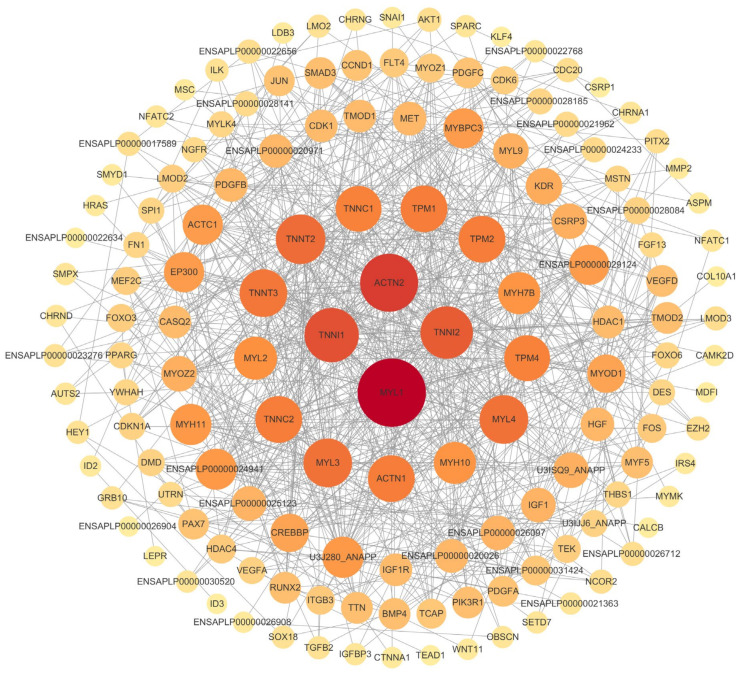
Protein–protein interaction network diagram. Darker colors indicate greater numbers of neighbor nodes.

To investigate interactions among the DEGs, we constructed protein–protein interaction networks using STRING. This network approach enabled a quick and intuitive understanding of the complex gene regulatory networks involved in skeletal muscle development (Figure 6). Within this network, MYL1, ACTN2, TNNI1, and TNNI2 were identified as central genes due to their high connectivity, suggesting crucial roles within the regulatory frameworks.

### 3.9. RT-qPCR Validation

**Figure 7:**
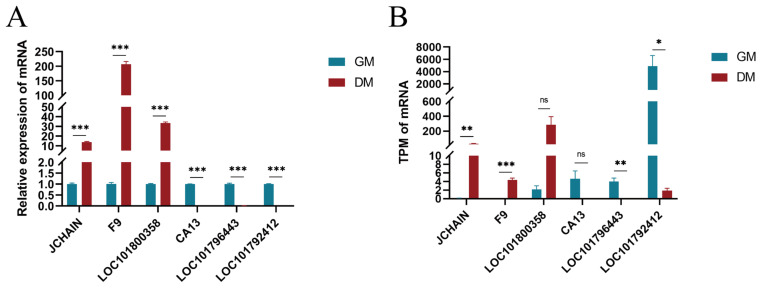
RT-qPCR validation of the sequencing data. (**A**) Relative expression of mRNAs in GM and DM groups using RT-qPCR; (**B**) TPM of mRNAs in GM and DM groups in the ONT sequencing. TPM: Transcripts per million. Values are presented as the mean ± SEM. * indicates *p* < 0.05, and ** indicates *p* < 0.01, *** indicates *p* < 0.001. GM indicates the proliferating myoblast group. DM indicates the differentiated myoblast group.

To validate the accuracy of our sequencing data, we randomly selected six genes (JCHAIN, F9, LOC101800358, CA13, LOC101796443, LOC101792412) from the pool of 3653 differentially expressed genes for validation through RT-qPCR. The experimental results supported the sequencing findings, strengthening the credibility of our transcriptomic data (Figure 7).

## 4. Discussion

Meat production holds significant economic importance in duck farming, making it essential to enhance both growth performance and meat quality [27]. The quality and quantity of skeletal muscle are critical indicators of meat quality [28], emphasizing the need for a deeper understanding of the mechanisms that underpin skeletal muscle growth and development. The development process is controlled by a range of genes, transcription factors, and non-coding RNAs such as lncRNAs, circRNAs, and microRNAs [29,30,31,32,33]. In duck embryos, primary skeletal muscle fibers begin to develop around day 6 of embryonic growth, with secondary fibers appearing between days 12 and 16. Research indicates that during the period from embryonic days 22 to 28, there is a noteworthy decline in the number of muscle fiber bundles and cross-sectional areas, which can lead to muscle atrophy [34]. Post-birth, the count of myofibers remains relatively constant, and muscle growth primarily occurs through hypertrophy. Thus, the processes of proliferation and differentiation in embryonic myoblasts significantly influence the levels of muscle mass achieved by poultry birds after birth [35]. Our study utilized full-length transcriptome sequencing to investigate leg muscle myoblasts isolated from duck embryos on day 13, focusing on their subsequent differentiation.

Our results indicated that lncRNAs, characterized by lengths exceeding 200 nucleotides and lacking protein-coding capability, were abundant in the myoblasts derived from duck embryos. Specifically, LincRNA and intronic lncRNAs emerged as the predominant forms, in contrast with the findings of studies on porcine dorsal longissimus muscle [36]. LncRNAs can influence skeletal muscle development through various mechanisms, including cis- and trans-regulatory interactions with target genes [37], functioning as antisense transcripts [38], acting as molecular sponges for microRNAs [39], or interacting with proteins to modulate their activity [40]. Furthermore, we have identified a novel lncRNA (ENSAPLG00000001939.t1) that may be derived from *TCF3*. Previous studies have shown that *TCF3* is a major regulator of muscle growth and homeostasis in pubertal heifers [41]. This lncRNA regulates *MYF5*, *ACTA2*, and *CDK1* by acting in trans. Subsequent studies could focus on the lncRNA. From our analysis, we predicted a total of 2332 lncRNAs, many of which are linked to skeletal muscle development, underscoring their potential role in regulating muscle growth in duck embryos.

Additionally, AS is a complex biological mechanism that contributes significantly to the intricacy of the transcriptomes found in multicellular eukaryotes [42]. Studies have shown that approximately 40% of protein modifications arise from AS, allowing a single mRNA precursor to produce diverse mRNA variants that encode proteins with distinct functions or structures [19]. It has been shown that certain muscle-development-related genes have AS events in different muscle tissues of different chicken breeds [43]. In a recent study, two isoforms of splicing factor *TRA2B* play distinct roles in myogenic differentiation by triggering the AS of *TGFBR2* to regulate canonical TGF-β signaling cascades differently [44]. In our research, we observed a high occurrence of skipped exon and alternative first exon events among differentially expressed splice variants, highlighting their importance during the proliferation and differentiation of duck embryo myoblasts.

GO enrichment analysis identified several genes—including *MYOM3*, *MYL2*, *SAP30, MYF5*, and *ACTN1*—that are associated with actin binding and muscle structural development. *MYOM3*, a newly recognized member of the myomesin family, is vital for maintaining myofibril integrity, particularly within the M-band, where it stabilizes myofilaments [45,46,47]. Previous studies have established connections between *MYOM3* and attributes of meat quality and muscle morphology [48,49], and have highlighted its significance in embryonic muscle fiber development [50]. Similarly, *MYL2*, part of the myosin light chain family, is crucial for muscle growth and contraction [51], possessing a promoter that operates specifically in skeletal muscle [52,53]. Furthermore, in muscle tissue, *SAP30* serves as a component of the histone deacetylase complex and acts as a transcriptional regulator [54]. Myogenic regulatory factors (MRFs), comprising transcription factors such as MyoD, Myogenin, Myf5, and Myf6, are essential for controlling myoblast proliferation and differentiation, directly impacting muscle fiber size and quantity [55,56]. Deficiencies in maternal nutrition have been shown to adversely affect the expression levels of these MRFs, reinforcing the necessity for adequate nutrition during embryonic development [55]. Moreover, *ACTN1*, which belongs to the spectrin superfamily, plays a critical role in stabilizing myofibrillar actin filaments [57]. Its downregulation during normal myoblast differentiation reflects its dynamic involvement in the processes governing muscle development [58]. Our findings also highlight the complex regulation of skeletal muscle development via various signaling pathways, including the Wnt, Notch, and mTOR pathways [59], all of which showed significant enrichment in our data. Notably, our study identified 2189 novel genes (Table S11), providing new directions for future research.

The protein–protein interaction network identified *MYL1* as a central gene involved in processes such as calcium binding, muscle structure maintenance, and myofilament sliding [60]. Importantly, research has established *MYL1*′s role in muscle fiber differentiation, particularly in zebrafish, where it functions as an early marker of rapid muscle cell differentiation [61]. Moreover, this study suggests that *MYL1* could be a promising candidate gene for improving meat quality, further supporting the notion that specific genes play pivotal roles in muscle development among chickens [62].

## 5. Conclusions

In summary, this study successfully utilized the ONT platform to construct the full-length transcriptome of duck embryo myoblasts, uncovering 5797 novel transcripts. Our analysis highlighted a substantial number of alternative splicing events, emphasizing the complexity of gene expression and its integral role in diverse cellular functions. An enrichment analysis revealed several key genes linked to skeletal muscle growth and development, particularly *MYOM3*, *MYL2*, *MYL1*, *TNNI2*, and *ACTN2*. These genes are strong candidates for regulating the proliferation and differentiation of duck embryo myoblasts. Overall, our findings can be used for further exploration in the context of duck skeletal muscle differentiation and growth, laying the foundation for future research in this field.

## Figures and Tables

**Table 1:** Primers for real-time quantitative PCR.

Gene ID	Gene Symbols in NCBI	Gene Description	Sequence (5′-3′)	Tm (°C)	Product Length (bp)
ENSAPLG00000024464	*JCHAIN*	immunoglobulin J chain	F:CATACGCATCACGGTCC R:GCCATGCGGTAGACAAAA	55.0	87
ENSAPLG00000010425	*F9*	coagulation factor IX	F:CGGATCCATCGTCAACGAGA R:TGTTGTATTCACCTGCCACG	59.0	92
ENSAPLG00000002251	*LOC101800358*	extracellular fatty acid-binding protein-like	F:ACCAGAGGGGTGTAGGAGAC R:CTATCGCCTCCCTCTGAGAC	59.0	84
ENSAPLG00000016183	*CA13*	carbonic anhydrase 13 isoform X2	F:AGTATGACCCCTCTCTCCGT R:CCCGGTCAGCACTGATTTG	59.0	128
ENSAPLG00000016444	*LOC101796443*	cystine/glutamate transporter isoform X1	F:GAGACCCTGGAGAGGATGTTC R:TCTTGCCATGGGCCTTGAT	59.0	103
ENSAPLG00000016406	*LOC101792412*	hemoglobin subunit alpha-A	F:CTGCTGTCATGTTAATGTTCCCT R:CCTATGAAGAGCCATCGGGA	59.0	103
ENSAPLG00030031700	*GAPDH*	glyceraldehyde-3-phosphate dehydrogenase	F: AAGGCTGAGAATGGGAAAC R: TTCAGGGACTTGTCATACTTC	60.0	254

**Table 2:** Full-length sequence statistics.

Sample Name	Mean Length (bp)	N50 (bp)	Max Length (bp)
GM1	1353.03	1970	19,387
GM2	1488.22	2113	21,494
GM3	1437.33	2101	21,354
DM1	1392.21	1933	20,896
DM2	1421.12	1971	21,953
DM3	1423.39	1978	21,434
Average	1419.22	2011	21,086

**Table 3:** Number of full-length reads and full-length reads mapped on genome.

Sample Name	Number of Clean Reads	Number of Full-Length Reads	Full-Length Percentage (FL%)	Mapped Reads	Mapped Rates Percent (%)
GM1	3,749,774	2,824,768	75.33	2,676,745	94.76
GM2	3,489,276	2,898,884	83.08	2,502,554	95.7
GM3	3,543,096	2,905,954	82.02	2,538,497	95.18
DM1	3,725,557	2,885,824	77.46	2,748,907	95.26
DM2	3,762,645	2,898,884	77.04	2,776,026	95.76
DM3	3,725,133	2,905,954	78.01	2,782,866	95.76

**Table 4:** Novel transcript annotation information.

Item	Count	Percentage (%)
All	5797	100.00
Annotation	1229	21.20
KEGG	997	17.20
Pathway	557	9.61
Nr	1197	20.65
Uniprot	1216	20.98
GO	591	10.19
KOG	25	0.43
Pfam	872	15.04

**Table 5:** Type and quantity of alternative splicing.

Types of Alternative Splicing	Differential Variable Splicing Quantity	Significant Differential Variable Splicing Quantity
Alternative 3′ splice site (A3)	70	8
Alternative 5′ splice site (A5)	83	6
Alternative first exon (AF)	576	57
Alternative last exon (AL)	353	36
Mutually exclusive exon (MX)	60	6
Retained intron (RI)	165	15
Skipping exon (SE)	939	75

**Table 6:** Type and number of SSRs identified.

Item	Number
Total length of sequence examined (bp)	85,085,181
Total number of SSR	28,677
Total length of SSR (bp)	46,317
Relative abundance (SSR/Mb)	337.04
Relative density (bp/Mb)	544.36
Number of SSR containing sequences	15,739
Number of sequences containing more than 1 SSR	6588

## Data Availability

We have deposited the raw sequence data in this paper into the Genome Sequence Archive (GSA). The accession number assigned to these datasets is GSA: CRA019664. These datasets can be publicly accessed at https://ngdc.cncb.ac.cn/gsa (accessed on 15 October 2024).

## Supplementary Materials

The following supporting information can be downloaded at: https://www.mdpi.com/article/10.3390/vetsci11120601/s1, Table S1: A3_gene.dpsi.add_info; Table S2: A5_gene.dpsi.add_info; Table S3: AF_gene.dpsi.add_info; Table S4: AL_gene.dpsi.add_info; Table S5: MX_gene.dpsi.add_info; Table S6: RI_gene.dpsi.add_info; Table S7: SE_gene.dpsi.add_info; Table S8: GM_vs_DM.Cis.Significant.DE_results.add_info; Table S9: GM_vs_DM.Trans.Significant.DE_results.add_info; Table S10: GM_vs_DM_DE_results.add_info; Table S11 gene.TPM.matrix.add_info.
